# Persons with epilepsy have an elevated radiosensitivity, which may be mitigated by folic acid

**DOI:** 10.1007/s00415-025-13593-0

**Published:** 2026-01-08

**Authors:** Melissa Kleber, Lukas C. F. Kuhlmann, Anabel Knabe, Jennifer Lainer, Stefan Schwab, Hajo M. Hamer, Stefanie Corradini, Luitpold Distel, Jenny Stritzelberger

**Affiliations:** 1 Department of Radiation Oncology, Universitätsklinikum Erlangen, Friedrich-Alexander University of Erlangen-Nürnberg (FAU), Universitätsstraße 27, 91054 Erlangen, Germany; 2 Epilepsy Center, Dept. of Neurology, Friedrich-Alexander University of Erlangen-Nürnberg, Full Member of EpiCare, Schwabachanlage 6, D-91054 Erlangen, Germany

**Keywords:** Epilepsy, Radiosensitivity, Antiseizure medication, Valproate, Lacosamide, Folic acid

## Abstract

**Introduction:**

Patients undergoing radiotherapy for cancer usually have several other comorbidities and are taking various medications. Both of these factors can affect the individual radiation sensitivity of normal tissue. We therefore studied persons with epilepsy (PWE) to determine whether they had altered radiation sensitivity.

**Material and Methods:**

Blood samples were collected from 105 adult patients with epilepsy and compared to those of healthy individuals and oncological patients. The samples were irradiated ex vivo and analyzed by 3-color fluorescence in situ hybridization. In each patient, aberrations were analyzed in 200 metaphases of chromosomes 1, 2, and 4 and scored as breaks. Radiosensitivity was determined by mean breaks per metaphase (B/M) and compared to both healthy donors and oncologic patients.

**Results:**

Radiosensitivity (B/M) of the PWE (*n* = 105, B/M: 0.478) was clearly increased compared to healthy individuals (*n* = 209, B/M: 0.420) and oncological patients (rectal patients and breast cancer patients, *n* = 319, B/M = 0.442). Antiseizure medications tended to increase radiation sensitivity. The use of perampanel (0.505 B/M; *p* = 0.081) and lacosamide (0.521 B/M; *p* = 0.014) led to a clear increase in radiation sensitivity. Male PWE (0.502B/M, *p* = 0.004) were distinctly radiosensitive which may be potentially explained by the fact that women were recommended to take high doses of folic acid according to German guidelines at the time of the study. Factors like seizure frequency, epilepsy duration, polytherapy and comedication did not contribute to increased radiosensitivity.

**Conclusion:**

PWE were clearly increased radiosensitive compared to healthy individuals and oncological patients. Male PWEs were found to be more sensitive than female PWEs. This may be due to their higher intake of folic acid, a substance which can protect against radiation.

**Supplementary Information:**

The online version contains supplementary material available at 10.1007/s00415-025-13593-0.

## Key points

**Table:** 

We analyzed ex-vivo radiosensitivity in 105 persons with epilepsy (PWE)
Compared to healthy individuals and patients with oncological diseases, PWE showed significantly higher radiation sensitivity
Male patients were more radiosensitive than females, possibly because women were taking high folic acid doses, which may offer radioprotective effects.

## Introduction

The challenge of radiotherapy is that it must eliminate cancerous cells while preserving the surrounding normal tissue. However, a variety of confounding factors can influence normal tissue tolerance. One such parameter is the patient's individual radiosensitivity. Radiotherapy-associated adverse effects not only impact patient’s quality of life but also potentially lead to discontinuation of therapy and even life-threatening conditions [1, 2]. By predicting individual radiosensitivity and adjusting the dosing regimen, side effects and adverse events could be avoided. Using chromosomal aberrations of blood lymphocytes to predict individual radiosensitivity has the benefit of addressing chromosomal aberrations as a late endpoint in the process of radiation damage. Chromosomal aberrations reflect a cell's ability to recover from and process DNA damage, including DNA damage repair, proper signal transduction mediation, appropriate cell cycle control, and induction of cell death if necessary [3–5]. Chromosome analysis identifies and scores chromosomal aberrations according to the number of underlying DNA double-strand breaks. The resulting value, breaks per metaphase, indicates how sensitive patients are to ionizing radiation [6, 7].

With 70 million people worldwide affected, epilepsy is a common disease. The relationship between epilepsy and cancer has been the subject of debate for a considerable period of time [8, 9]. In addition to epilepsy itself being a risk factor for cancer in persons with epilepsy (PWE) due to diagnostic procedures or the lifestyle of PWE, the propensity of antiseizure medication (ASM) to promote or protect against cancer has been discussed in several previous animal and epidemiological studies with contradictory results [10, 11]. ASM may alter oxidative stress responses and tissue repair, which could potentially affect radiosensitivity.

Persons with epilepsy (PWE) may require radiotherapy (RT) in various situations. A significant proportion of adult-onset epilepsy cases are caused by structural lesions in the brain, particularly gliomas, metastases, and meningiomas. RT is a standard component of the treatment of these conditions. In select cases, radiosurgery may be considered for specific epileptogenic lesions. It is valuable to assess individual radiosensitivity in order to anticipate both therapeutic efficacy and the risk of radiation-induced complications, including necrosis, cognitive decline, and seizure exacerbation. Furthermore, as with the general population, PWE may develop cancers outside the central nervous system.

Our previous work identified various diseases and underlying conditions that render patients more sensitive to irradiation, for example autoimmune diseases like Lupus Erythematodes [12], genetic diseases like Phelan-McDermid Syndrome [13], tuberous sclerosis [14] and drugs like valproic acid [15]. The objective of this study was to ascertain whether epilepsy as a condition itself renders patients more sensitive to radiation, and if so, which other factors may contribute to this sensitivity in PWE.

## Material and methods

### Patient cohorts blood samples

Venous blood samples for radiosensitivity testing were collected from 105 patients with confirmed epilepsy diagnoses during their stays at the Epilepsy Center in Erlangen, in both inpatient and outpatient settings. Sampling took place between February 2019 and September 2023. For this study, individuals with other medical conditions or taking drugs suspected to increase radiosensitivity, such as chloroquine, efavirenz, nelfinavir, vandetanib, metformin, or immunotherapy, were excluded from this study, as were individuals with malignant neoplasms. Some of this data has been previously published [14, 15].

To define average and increased radiation sensitivity, the results of 209 healthy control subjects were compared with those of 221 patients with rectal cancer and 121 patients with breast cancer. These results had previously been published by the Department of Radiation Oncology at Erlangen University Hospital [7, 12]. This study was approved by the Ethics Committee of Friedrich-Alexander University Erlangen-Nuremberg (approval no. 21_19B). All patients gave written informed consent [7, 16, 17].

### Fluorescence in Situ-Hybridization assay (FiSH)

A volume of 9 mL of venous blood was collected from each patient into a heparin tube (Ammonium -Heparin, Sarstedt, Nümbrecht, Germany). The blood sample was divided into two equal parts. One half was irradiated in a tissue block with a dose of 2 Gray (Gy) by a 6-MV linear accelerator (Versa HD, Elekta, Stockholm, Sweden). The dose of 2 Gy corresponds to a standard dose in radiotherapy. The other half remained unirradiated and was used to assess the baseline level of chromosomal aberrations in each patient. This background level was then subtracted from the chromosomal aberrations that were irradiated at 2 Gy. A culture medium composed of RPMI, 1% penicillin/streptomycin, 15% fetal calf serum and 6% phytohemagglutinin was prepared and distributed across 8 to 10 culture flasks. In 4–5 of these flasks, 1 mL of irradiated blood was added, while 1 mL of unirradiated blood was added to the remaining 4–5 flasks. The flasks were incubated upright at 37 °C for 48 h [18]. Entry of lymphocytes into the cell cycle was stimulated by phytohemagglutinin [19], and after 48 h, the cell cycle was arrested in metaphase by the addition of colcemid (Gibco, USA). Chromosomes from the cultured lymphocytes were prepared on slides following a standard protocol. Chromosomes 1, 2, and 4 were labeled in red, green, and yellow by using fluorescence dyes. After this the chromosomes were counterstained blue with DAPI. The three-color fluorescence in situ hybridization was used as previously described [20, 21].

### Determination of chromosomal aberrations

Image acquisition was performed using a fluorescence microscope (Zeiss, Axioplan 2, Germany) and an automated metaphase scanner (Metafer 4, Germany). The labeled chromosomes 1, 2, and 4 were then analyzed for chromosomal aberrations. A specialized image analysis software (Biomas, Erlangen, Germany) was used to evaluate the aberrations. The software supports manual evaluation of the images and automatically exports the results into Excel spreadsheets (Excel, Microsoft Corporation, Redmond, WA, USA). The potential number of DNA double-strand breaks was assigned to each aberration type according to the classification by Savage and Simpson [22]. Breaks and deletions were each scored as one break. Translocations, dicentrics and rings as two. Insertions as three. For complex aberrations, the assigned break number reflected the minimal theoretical requirement for their formation. The B/M value of the background sample was subtracted from that of the irradiated sample to determine the B/M value induced by the 2 Gy dose. The number of breaks per metaphase (B/M) was calculated from an average of 188 metaphases for irradiated samples and an average of 101 metaphases for unirradiated samples.

### Statistics

Graph Pad Prism (GraphPad Software, San Diego, California, USA), SPSS (version 26.0, IBM, NY, USA, Armonk), R Studio, BioRender (Toronto, Ontario, Canada), and Microsoft Excel 365 (Microsoft Corporation, Redmond, Washington, USA) were used for statistical calculations and data visualization. Kruskal–Wallis-Test was used to compare the different cohorts. In the post-hoc analyses, T-test (*n* > 30) or non-parametric Mann–Whitney U-test (*n* < 30) was used to compare the different cohorts for significant differences. To assess potential correlations between epilepsy-associated characteristics with radiosensitivity, we used univariate and multivariate analyses. For these analyses, we dichotomized numerical data using the mean or median, to create equal groups. The level of significance was set at 5%.

## Results

### Cohorts

In order to determine whether persons with epilepsy (PWE) are more sensitive to radiation than the general population, we compared them with 208 healthy individuals, 221 patients with rectal cancer and 126 patients with breast cancer. With a mean age of 36.6 years, the PWE cohort was significantly younger than the other cohorts (*p* < 0.001) (Table 1). The PWE cohort had a higher percentage of males than the healthy control group, though this was lower than in the oncological groups. 20% of PWE were diagnosed with generalized epilepsy and 62.9% with focal epilepsy. Fourteen patients had developmental epileptic encephalopathy, thirteen of whom had tuberous sclerosis (12.4% of all PWE). The average duration of epilepsy was 19.0 years and the average seizure frequency was 18.5 per month (Table 2).

**Table 1:** Age and sex of the respective cohorts

	Age (years)					Sex		
	n	Mean	SD	Min	Max	n	Male (%)	Female (%)
Persons with epilepsy	105	36.6	15.7	18	84	105	60 (57.1)	45 (42.9)
Healthy individuals	208	50.1	17.7	9	81	208	89 (42.8)	119 (57.2)
Oncological patients								
Rectal cancer	221	62.8	12.3	23	87	221	155 (70.1)	66 (29.9)
Breast cancer	126	56.6	12.6	28	91	126	0 (0)	126 (100)

**Table 2:** Additional characteristics of the PWE regarding their epilepsy diagnosis

	n	%	Mean	SD	Min	max
Epilepsy syndromes						
Genetic generalized epilepsy	21	20.0	–	–	–	–
Focal epilepsy	66	62.9	–	–	–	–
Tuberous sclerosis	13	12.4	–	–	–	–
Dravet syndrome	1	1.0	–	–	–	–
Epilepsy of unknown syndrome	4	3.8	–	–	–	–
Duration of epilepsy (years)	–	–	19.0	15.8	0	60
Seizure frequency per month	–	–	18.5	58.5	0	500
Tonic–clonic seizures	73	69.5	–	–	–	–
Seizure frequency tonic–clonic seizures per month	–	–	0.5	1.5	0	12
Antiseizure medication (ASM)						
Levetiracetam	21	20.0	–	–	–	–
Lamotrigin	19	18.1	–	–	–	–
Lacosamid	16	15.2	–	–	–	–
Vaproic acid	30	28.6	–	–	–	–
Perampanel	43	41.0	–	–	–	–
Oxcarbazepin	6	5.7	–	–	–	–
Topiramat	6	5,7	–	–	–	–
Benzodiazepine	7	6,7	–	–	–	–
Others	20	19.0	–	–	–	–
Number of ASM					0	5
None	35	33.3	–	–	–	–
1 ASM	12	11.4	–	–	–	–
2 ASM	32	30.5	–	–	–	–
3 or more	26	24.8	–	–	–	–
Number of Comedication (no ASM)					0	7
None	79	75.2	–	–	–	–
1	19	18.2	–	–	–	–
2	5	4.8	–	–	–	–
3 or more	2	2.0	–	–	–	–

### Comparison of radiosensitivity between PWE, healthy controls and oncological patients

**Fig. 1:**
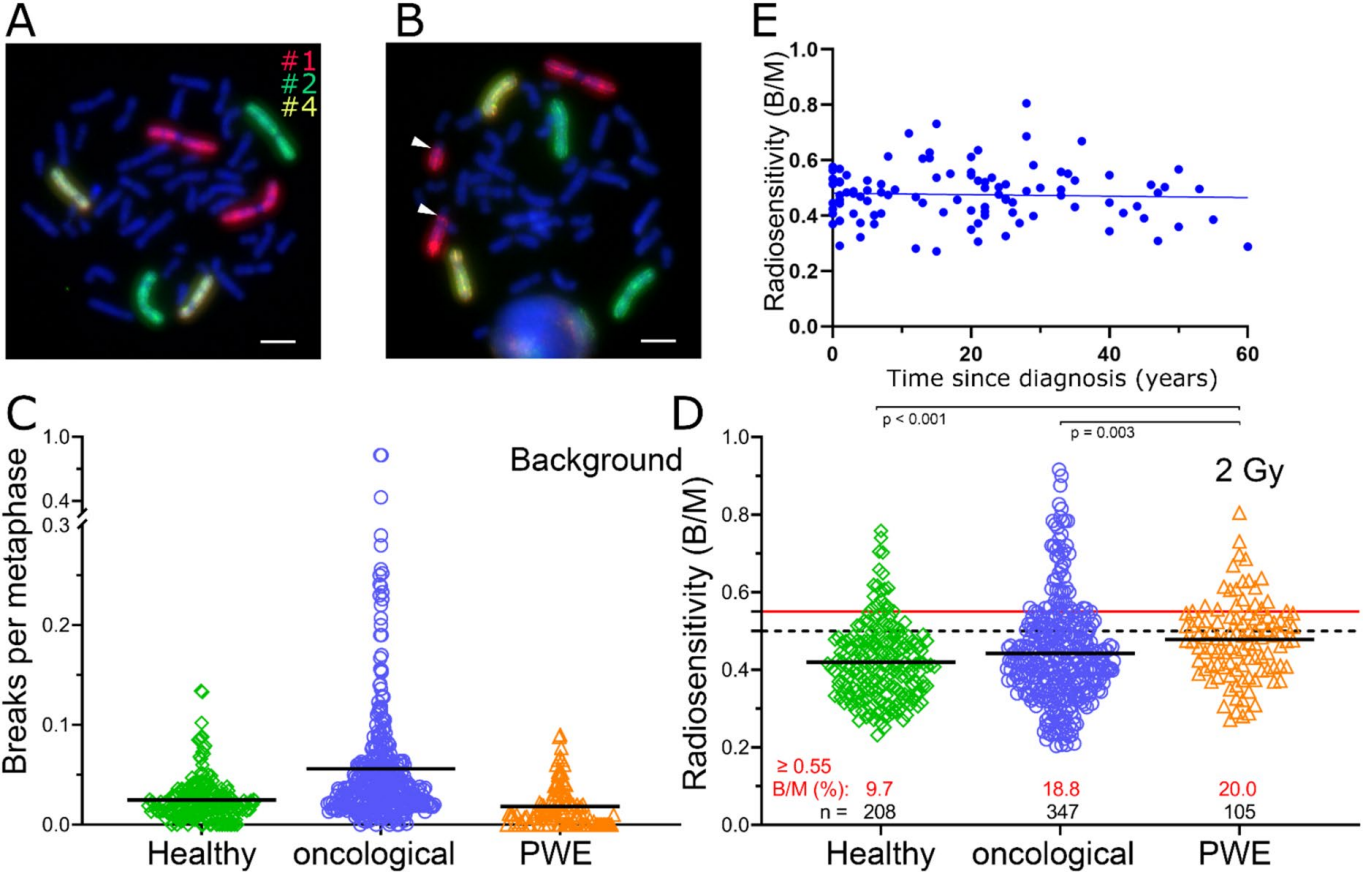
Comparison of radiation sensitivity in healthy individuals, cancer patients, and patients with epilepsy (PWE) was conducted using three-color in situ hybridization. **A** Metaphase with chromosomes 1, 2, and 4 (red, green, and yellow, respectively) without chromosomal aberrations. **B** Metaphase with a translocation, indicated by the arrowheads. Scale bars indicate 10 µm. **C** Background of chromosomal aberrations expressed as breaks per metaphase. **D** Chromosomal aberrations after ex vivo irradiation with 2 Gy IR. The black solid lines indicate the mean value of the cohorts. The black dashed line indicates the 0.5 B/M threshold at which increased radiosensitivity is indicated. The solid red line indicates 0.55 B/M, the point at which a radiotherapy dose adjustment should be made. The number of individuals is shown in black, and the percentage of patients with a B/M greater than 0.55 is shown in red. **E** Radiosensitivity (B/M) in relation to the duration (in years) since the epilepsy diagnosis. The solid blue line indicates the correlation

We assessed radiosensitivity using three-color fluorescence in-situ hybridization (FISH) (Fig. 1A, B). Breaks per metaphase (B/M) correspond to the weighted chromosomal aberrations necessary for forming the respective aberration. Background values were highest in the oncological cohort (*p* < 0.001), slightly lower in healthy individuals (*p* = 0.011), and lowest in the PWE cohort (Fig. 1C). However, the value of 2 Gy-induced chromosomal aberrations expressed as B/M was significantly higher in the PWE cohort (mean value: 0.478) than in both the healthy control group (0.420; *p* < 0.001) and the oncological patient group (0.442; *p* = 0.003) (Fig. 1D).

Therefore, we examined various disease-related parameters. There was no clear association between time since epilepsy diagnosis and radiosensitivity (B/M) (R^2^ = 0.002). The slope was not significantly different from zero (*p* = 0.675) (Fig. 1E). Additionally, there was no linear relationship between seizure frequency (R^2^ = 0.009, *p* = 0.342), tonic–clonic seizure frequency (R^2^ = 0.001, *p* = 0.751), or the time between the last seizure and blood sampling (R^2^ = 0.005, *p* = 0.552) and radiosensitivity (B/M) (Supplementary figure S1).

### Evaluation of potential impact factors on radiosensitivity

Due to the high incidence of chromosomal aberrations observed in PWE, our study aimed to investigate potential contributing factors. To this end, we conducted a subgroup analysis within the epilepsy patient cohort.

### Demographic factors

**Fig. 2:**
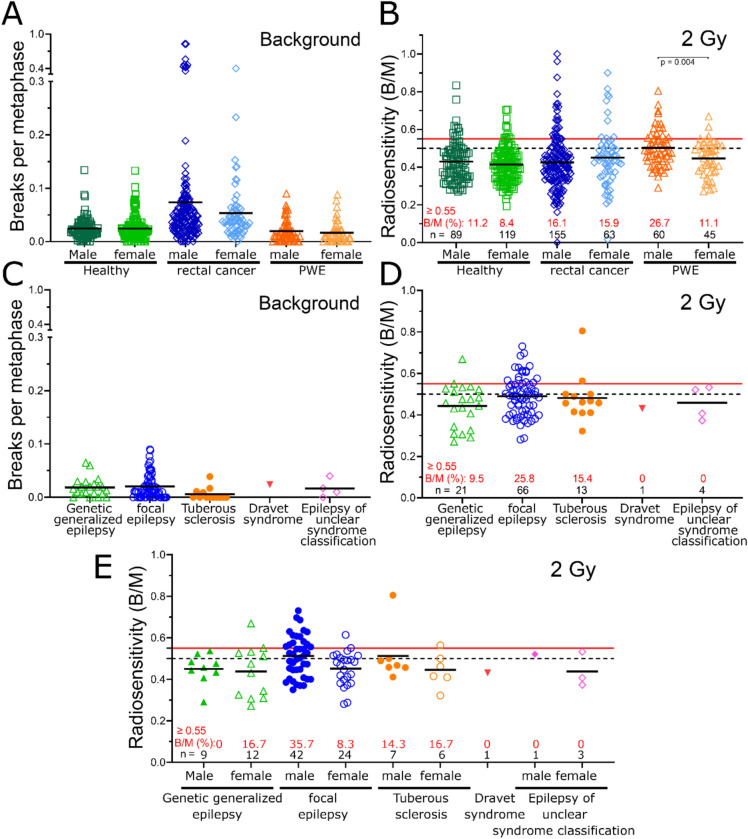
Radiation sensitivity in healthy individuals, rectal cancer patients, and PWE, with all subgroups additionally stratified by sex (male or female). **A** Background of chromosomal aberrations expressed as breaks per metaphase. **B** Chromosomal aberrations after ex vivo irradiation with 2 Gy IR. Radiation sensitivity in PWE in dependence of the epilepsy subtype. Comparison of genetic generalized epilepsy, focal epilepsy, tuberous sclerosis, Dravet syndrome and epilepsy with an unclear syndrome classification. **C** Background of chromosomal aberrations expressed as breaks per metaphase. **D** Chromosomal aberrations after ex vivo irradiation with 2 Gy IR. **E** Comparison of genetic generalized epilepsy, focal epilepsy, tuberous sclerosis, Dravet syndrome, and epilepsy of unclear syndrome classification. All subgroups are additionally stratified by sex (male or female). Chromosomal aberrations after ex vivo irradiation with 2 Gy IR. Black solid lines indicate the mean value of the cohorts. The black dashed line indicates the 0.5 B/M threshold at which increased radiosensitivity is indicated. The solid red line indicates 0.55 B/M, at which point a radiotherapy dose adjustment should be made. The number of individuals is shown in black, and the percentage of patients with a B/M greater than 0.55 is shown in red

Considering the average age, the PWE cohort had a mean age of 36.6 years, making them the youngest compared to healthy controls (50.1 years), rectal cancer patients (62.8 years), and breast cancer patients (56.6 years). Background aberrations did not differ between males and females in the healthy control group (male: 0.0247 B/M; female: 0.0245 B/M; *p* = 0.948), the PWE cohort (male: 0.0195 B/M; female: 0.016 B/M; *p* = 0.464), or the rectal cancer cohort (male: 0.074 B/M; female: 0.053 B/M; *p* = 0.111) (Fig. 2A). After irradiation with 2 Gy, radiosensitivity was not different between the male and female subgroups in either the healthy control group (male: 0.429 B/M; female: 0.414 B/M; *p* = 0.286) or the rectal cancer group (male: 0.425 B/M; female: 0.450 B/M; *p* = 0.2516). However, in clear contrast, the PWE cohort had notably higher radiosensitivity in the male subgroup (0.502 B/M) than in the female subgroup (0.446 B/M) (*p* = 0.004) (Fig. 2B). These findings suggest that there are no general sex-specific differences in radiosensitivity. Instead, there are epilepsy-specific causes of the difference in radiosensitivity between males and females. Therefore, we focused our further subgroup analysis on epilepsy-related factors with a special focus on sex distribution.

Factors related to the disease, such as the presence of epilepsy syndrome, the duration of the condition and the frequency of seizures.

The background in the tuberous sclerosis subgroup (mean B/M value: 0.0058) was lower than in the genetic generalized epilepsy subgroup (0.019; *p* = 0.008) and the focal epilepsy subgroup (0.021; *p* = 0.004). There was no difference between the tuberous sclerosis subgroup and the epilepsy of unclear syndrome subgroup (0.017 B/M; *p* = 0.1576) (Fig. 2C). After irradiation with 2 Gy, chromosomal aberrations were slightly increased in focal epilepsy patients (0.490; *p* = 0.0983) and in the tuberous sclerosis subgroup (0.482; *p* = 0.490) than in patients with genetic generalized epilepsy (0.443; *p* = 0.0983) (Fig. 2D). For focal epilepsy, the increase in radiosensitivity was more pronounced in the male subgroup in focal epilepsy (males 0.513 B/M, females 0.451, *p* = 0.021). There were no sex-related differences in the tuberous sclerosis subgroup and the genetic generalized epilepsy subgroup (TS male: 0.512 B/M; female: 0.445 B/M; *p* = 0.469; GGE male: 0.450 B/M; female: 0.438; *p* = 0.917) (Fig. 2E).

### Effect of antiseizure medications on radiation sensitivity

**Fig. 3:**
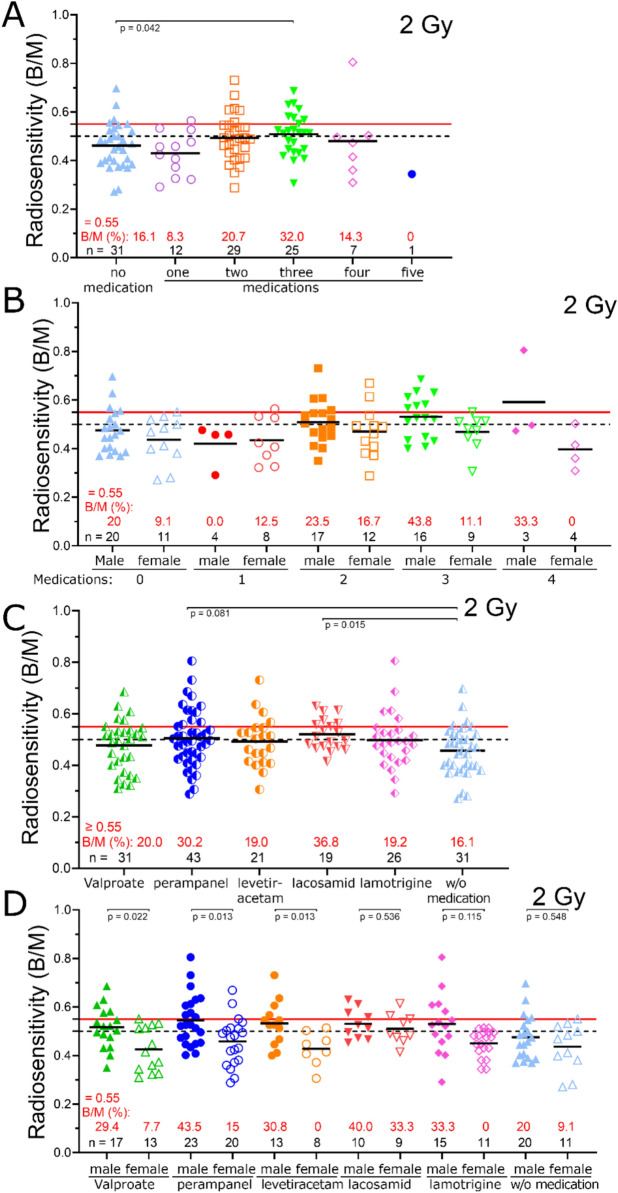
**A** Comparison of radiation sensitivity in PWE taking no medication or 1 to 5 medications simultaneously. Chromosomal aberrations after ex vivo irradiation with 2 Gy IR. **B** Comparison of radiation sensitivity in PWE taking no medication or 1 to 4 medications simultaneously. All subgroups stratified by sex (male, female). Chromosomal aberrations after ex vivo irradiation with 2 Gy IR. **C** Comparison of radiation sensitivity in PWE taking various medications, including valproate, perampanel, levetiracetam, lacosamid, lamotrigine and PWE without medication using three-color in situ hybridization. Chromosomal aberrations after ex vivo irradiation with 2 Gy IR. **D** Comparison of radiation sensitivity in PWE taking various medications, including valproate, perampanel, levetiracetam, lacosamid, lamotrigine and PWE without medication using three-color in situ hybridization. All subgroups stratified by sex (male, female). Chromosomal aberrations after ex vivo irradiation with 2 Gy IR. Black solid lines indicate the mean value of the cohorts. The black dashed line indicates the threshold of 0.5 B/M from which increased radiosensitivity is indicated. The solid red line indicates 0.55 B/M, at which point a dose adjustment in radiotherapy should be made. The number of individuals is given in black and the percentage of patients having a B/M > 0.55 is given in red

We investigated whether the use of antiseizure medications (ASMs) influences radiosensitivity in PWE. Although PWE receiving ASMs tended to exhibit higher mean B/M values (0.493) than those not on medication (B/M = 0.463), this difference was not significant (*p* > 0.206). To assess the impact of polytherapy, we analyzed radiosensitivity based on the number of concurrently used ASMs. Mean B/M values were 0.430 for patients on one ASM, 0.493 B/M for two, 0.509 B/M for three, 0.480 B/M for four, and 0.344 B/M for five, compared to 0.463 B/M in those not taking ASMs. A significant increase in radiosensitivity was observed only in patients taking three ASMs compared to those on no medication (*p* = 0.042), while other comparisons did not reach significance (*p* > 0.217) (Fig. 3A).

We further examined sex-specific differences in radiosensitivity among PWE taking between zero and four ASMs. No significant differences were observed between males and females in any subgroup (*p* > 0.093). However, the mean B/M values for males were slightly higher across all groups, with the exception of those on monotherapy (Fig. 3B).

We then analyzed the influence of each ASM. PWE treated with perampanel (0.505 B/M; *p* = 0.081) or lacosamide (0.521 B/M; *p* = 0.014) exhibited increased radiosensitivity compared to the subgroup not taking medication (Fig. 3C). A sex-stratified analysis of drug-associated radiosensitivity revealed that the observed increase was primarily attributable to male patients (Fig. 3D).

### Comedication with folic acid

Since our analyses showed that none of the following could explain the increased radiosensitivity in male PWE: epilepsy syndrome, ASM intake, duration of epilepsy, or seizure frequency, we looked into comedication. First, we examined the number of comedications taken by the PWE in our study. PWE without comedication showed a mean B/M-value of 0.462. Those with one comedication 0.466 B/M, with two 0.489 B/M, with three 0.507 B/M and with four 0.482 B/M. In our study, there was also one patient who took five comedications, showing a B/M-value of 0.344 B/M, and another who took seven comedications, with a B/M-value of 0.326 B/M. We then compared the patients without comedication to those with different numbers of comedications (one to four) and found no significant difference (*p* > 0.095).

**Fig. 4:**
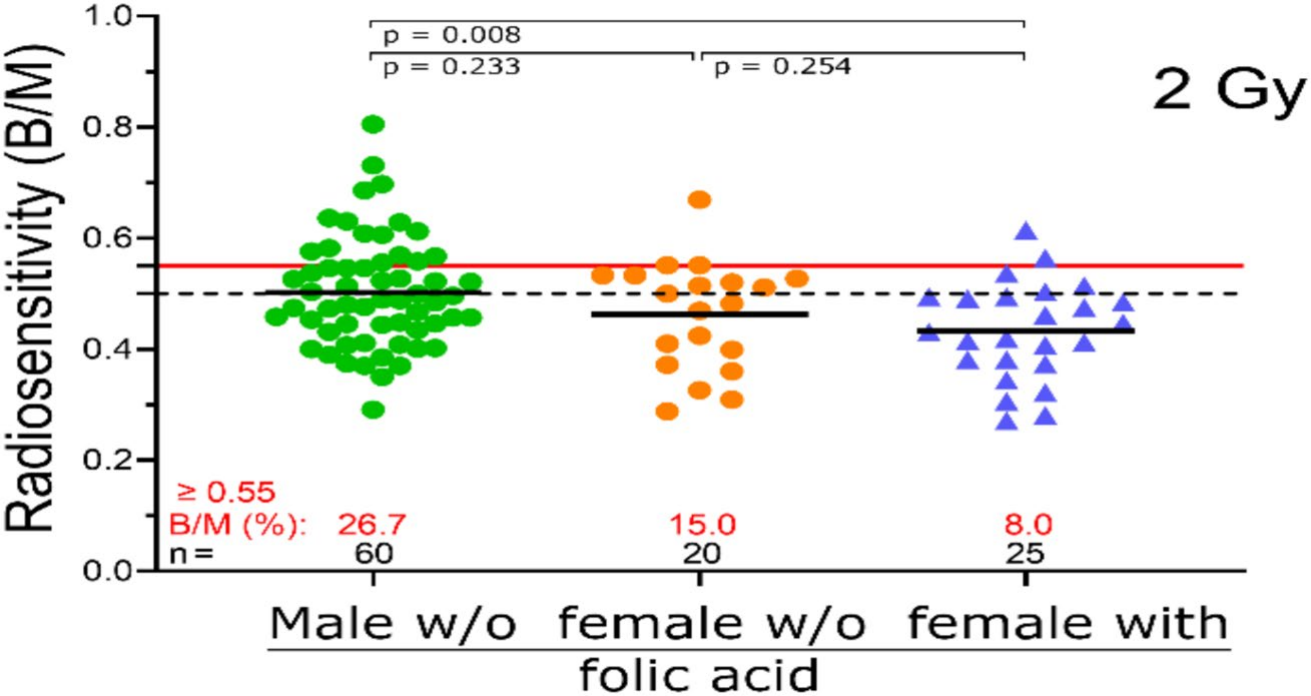
Radiation sensitivity is compared between male and female patients with epilepsy (PWE) who do not take folic acid and female PWE who do. Chromosomal aberrations after ex vivo irradiation with 2 Gy IR. The black solid lines indicate the mean value of the cohorts. The black dashed line indicates the 0.5 B/M threshold at which increased radiosensitivity is indicated. The solid red line indicates 0.55 B/M, the point at which a radiotherapy dose adjustment should be made. The number of individuals is shown in black, and the percentage of patients with a B/M greater than 0.55 is shown in red

One apparent difference between the male and female participants in our study was that women of childbearing age were recommended to take high doses of folic acid (up to 5 mg per day) according to German guidelines. First, we compared men and women who did not take folic acid. There was no significant difference in radiosensitivity between the two groups (male: 0.502 B/M; female: 0.462 B/M; *p* = 0.233). Next, we compared male patients who did not take folic acid with female patients who did. This comparison revealed a significant difference, with the group of females taking folic acid having clearly lower B/M values (0.431 B/M; *p* = 0.008). No significant difference was found when female patients who took folic acid were compared with those who did not (*p* = 0.254). However, lower B/M values were apparent in the group that took folic acid (Fig. 4).

## Discussion

Our study revealed that PWE were clearly more sensitive to ionizing radiation than healthy individuals or oncology patients, which is our key finding. Previous studies have demonstrated that oncological patients are more radiosensitive than healthy individuals [5]. Remarkably, PWE were even more sensitive than oncological patients. In our study, 20.0% of the PWE were at or above the 0.55 B/M threshold. From this level onwards, we would strongly recommend reducing the dose, as otherwise the risk of undesirable side effects increases.

Several studies have revealed that the analysis of radiation-induced chromosomal aberrations [3], especially fluorescence in-situ hybridization (FISH), is suitable for predicting radiosensitivity. This is because the B/M value corresponds to clinical radiotherapy side effects [12, 23].

PWE may require radiotherapy (RT) in various situations. It is valuable to assess individual radiosensitivity in order to anticipate both therapeutic efficacy and the risk of radiation-induced complications, including necrosis, cognitive decline, and seizure exacerbation. Testing for radiosensitivity could be important for certain patients to determine their risk and allow for personalized dosage adjustments. Reducing the radiation dose could decrease the likelihood of side effects in radiosensitive patients. However, since radiosensitivity testing is not yet clinically established, this dose reduction should be carried out very cautiously to avoid underdosing and, consequently, insufficient tumor treatment. We do recommend strict follow-up for these patients to detect potential late side effects [24].

We examined the background values of the different cohorts and observed that these were lowest in the PWE compared to oncological patients and the healthy controls. This finding may be explained by the younger age of the PWE cohort. The patient's background does not indicate radiosensitivity. Rather, it is determined by the patient's environment and whether radiotherapy was previously administered [7]. However, radiation sensitivity is already influenced by age. Over the course of life, the repair capacity of cells declines [25] and radiosensitivity increases continuously, making older individuals slightly more radiosensitive. However, this increase is subject to strong interindividual variation [16]. Since male PWE were more radiosensitive than women, which was a surprising finding, we conducted several sub analyses to investigate the underlying cause, since it is well known that sex alone does not influence radiosensitivity [16, 17], and we found no sex-related changes in radiosensitivity in either healthy individuals or cancer patients.

One possible explanation for the observed sex difference could have been that males were overrepresented in the subgroups for which we found non-significant trends for increased radiosensitivity like focal epilepsy, TS, or certain ASM, and that these factors, rather than sex, were the true driver of increased radiosensitivity. However, the higher radiosensitivity of males was consistently observed across all subgroups, while sex distribution was balanced throughout the subgroups, making this explanation unlikely. Instead, these findings suggest that the difference is more directly related to sex-specific factors. Another possibility is that ASMs are metabolized differently in women than in men. Although there are indications that metabolism is altered, there is no evidence of a significant increase in metabolism that could lead to reduced sensitivity to radiation by the ASMs [26, 27]. Another possibility is autoimmune diseases, which could lead to increased sensitivity to radiation. Unfortunately, we have no data on whether our PWEs suffered from these diseases. However, since women are at least twice as likely to have autoimmune diseases [28], it is unlikely that increased radiation sensitivity in men is caused by them. A potential contributing factor may be that female patients with epilepsy are recommended to take folic acid, which could modulate radiosensitivity. Up until 2023, German guidelines recommended female PWE to take high doses of 5 mg folic acid/day [29]. Indeed, female PWE who took folic acid were significantly less radiosensitive than male PWE and there was no difference between women who did not take folic acid and male PWE, indicating that folic acid may have been the underlying cause for the difference we observed. Radioprotective effects of antioxidants like vitamin C are well-known [30, 31]. It has also been shown that antioxidants during radiotherapy or chemotherapy in breast cancer patients can lead to a deterioration in disease-free survival and overall survival [32]. In a study on radiation therapy for head and neck tumors, α-tocopherol and β-carotene protected normal tissue but also led to increased local recurrence [33]. Folic acid modulates oxidative stress response [34] as well, and has been found to exert radioprotective effects by reducing cellular senescence in vitro [35]. Folic acid is an extremely good radical scavenger that scavenges almost all radicals induced by ionizing radiation. The reaction rates of folic acid with the most IR-induced reactive radicals OH· (9.6 × 10⁹ L·mol⁻^1^·s⁻^1^) and the hydrated electron (2.2 × 10^1^⁰ L·mol⁻^1^·s⁻^1^) are so high that diffusion is the only limiting factor in their reaction with folic acid [36]. This suggests that folic acid protects very well against IR. Animal experiments have shown that teratogenic effects can be counteracted by folic acid (8 mg/kg FA). Further studies in mice demonstrated protection of the ovaries (5 mg/kg FA) [37] and lungs (10 mg/kg FA) [35] in mice. Our study could be the first study to show a protective effect of folic acid against IR in humans. Compared to the mouse experiments, a significantly lower dose of 5 mg per day was taken, but this was taken over a long period of time so that a level of folic acid could build up in the tissue [38, 39]. Therefore, the association between folic acid supplementation in women and reduced radiation sensitivity is quite strong. No difference in radiation sensitivity between women and men has ever been found, and we were unable to identify any other factors. However, although female PWE are recommended to take folic acid, we did not have plasma folic acid levels to prove this hypothesis.

We did not identify specific disease- or therapy-related factors that could fully account for the observed increase in radiosensitivity of PWE compared to the control groups, although certain ASMs may play a contributory role. Notably, somatic morbidity is increased in PWE [40] which may partially be explained by biological or genetic factors [41]; a possible link between increased radiosensitivity and the elevated somatic morbidity in PWE may be considered. Radiosensitivity often reflects impaired DNA repair or increased oxidative stress, mechanisms that are also implicated in cardiovascular and metabolic disorders. Therefore, radiosensitivity may indicate broader systemic vulnerability rather than being limited to radiation-induced effects.

One strength of this study is that it represents a large cohort group with 105 PWE, which allows reliable conclusions to be drawn regarding radiosensitivity of this group. However, within the subgroups, sample sizes were relatively small, which may have obscured relevant associations. As our group has previously reported, patients receiving valproate displayed increased radiosensitivity, most likely due to already well-known radiosensitizing properties of VPA as an HDAC-inhibitor [42, 43]. We confirmed this trend [15]; however, the difference compared to patients not taking medication did not reach statistical significance, likely due to the limited number of valproate users (*n* = 30). Similarly, 25.8% of patients with focal epilepsy and 38.5% of patients with tuberous sclerosis had B/M values above the 0.55 threshold, suggesting increased radiosensitivity. However, these results did not reach significance, most likely due to the small subgroup sizes (TS *n* = 13, genetic generalized epilepsy *n* = 22, focal epilepsy *n* = 66). Additionally, PWE on ASM exhibited higher, albeit nonsignificant, B/M values than untreated PWE. Perampanel and lacosamide appeared to increase radiosensitivity in particular. As these drugs were not administered as monotherapy, we analyzed radiosensitivity in relation to the number of concomitant ASMs and found no linear correlation. Patients taking three ASMs displayed the highest radiosensitivity, while those taking more than three ASMs did not. Of note, PWE with polytherapy were overrepresented in our cohort since our institution is a tertiary epilepsy center with a high number of patients with drug-resistant epilepsies. Taken together, these observations should be interpreted with caution, as low case numbers within these groups limit the robustness of the conclusions.

## Conclusion

The study showed that PWE were more radiosensitive compared to healthy individuals and cancer patients. One factor that potentially may have decreased radiosensitivity was high-dose folic acid intake in female PWE. Further studies are needed to confirm these results, better predict which patients are affected, and analyze potential aggravating factors, such as medication. These studies should include radiosensitivity analyses of more patients, especially those with genetic epilepsy or rare cancer risk genes.

## Supplementary Information

Below is the link to the electronic supplementary material.

Supplementary file1 (PDF 965 KB)

## Data Availability

The datasets generated and/or analyzed in the present study are not publicly accessible but can be provided by the corresponding author upon reasonable request.
