# Supplementary material for: Persons with epilepsy have an elevated radiosensitivity, which may be mitigated by folic acid
Source: J Neurol. 2026 Jan 8;273(1):66. doi: 10.1007/s00415-025-13593-0 (PMC12783278; doi:10.1007/s00415-025-13593-0)
Supplement: Supplementary file 1 — Supplementary file1 (PDF 965 KB) [file 415_2025_13593_MOESM1_ESM.pdf]

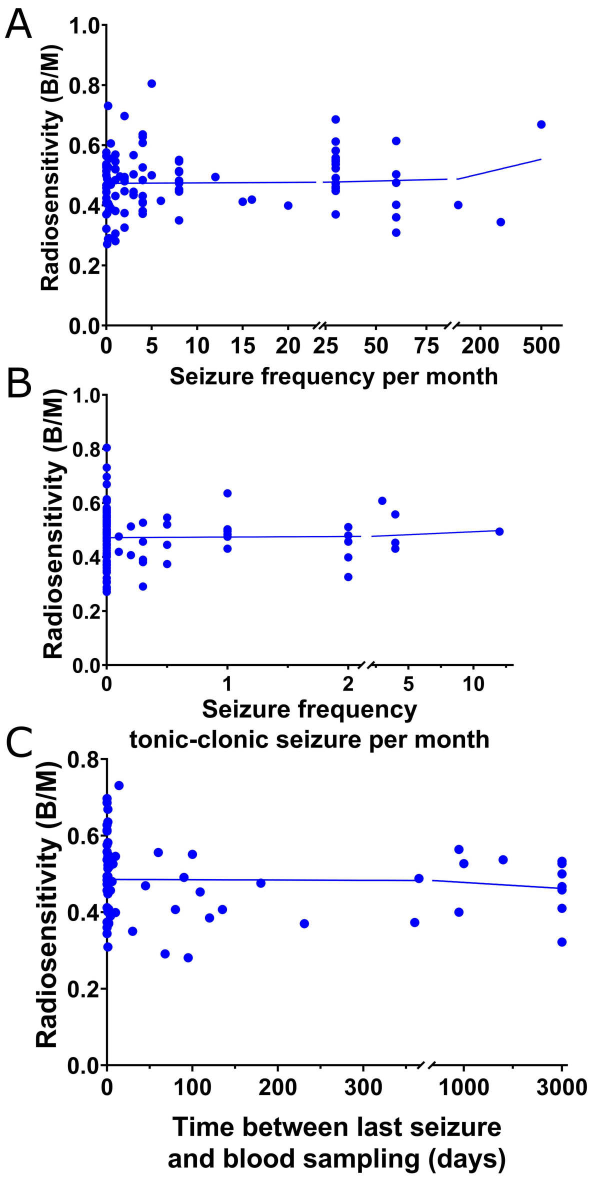

Supplementary figure 1 Radiosensitivity (B/M) in relation to the (A) seizure frequency per month, (B) seizure frequency of tonic-clonic seizures per month, (C) time between last seizure and blood sampling in days.
